# Kidney transplant survival in pediatric and young adults

**DOI:** 10.1186/1471-2369-12-54

**Published:** 2011-10-07

**Authors:** James A Kiberd, Phil Acott, Bryce A Kiberd

**Affiliations:** 1 Department of Medicine and Pediatrics, Dalhousie Medical School, Halifax, Nova Scotia, Canada

## Abstract

**Background:**

There is a perception that kidney transplant recipients transferred from pediatric centers to adult care have an increased risk of graft loss. It is not clear whether young adults transplanted in adult centers also suffer from high graft loss rates.

**Methods:**

We examined death censored graft survival in 3 cohorts of young patients transplanted at a single center. Pediatric (PED) patients transplanted at the pediatric center were compared to a cohort of young adults (YAD; age 18- < 25) and a cohort of adults (ADL; age 25-35).

**Results:**

In a multivariate Cox model for death-censored graft survival, PED survival was statistically similar to the YAD (HR 0.86, 95% CI 0.44, 1.7, p = 0.66), however the ADL cohort (HR 0.45, 95% CI 0.25, 0.82, p = 0.009) demonstrated better survival. Admitted non-adherence rates were not different among cohorts. Patients were transferred within a narrow age window (18.6 ± 1.0 age in years) but at a wide range of times from the date of transplantation (5.1 ± 3.5 years) and with a wide range of graft function (serum creatinine 182 ± 81 μmol/L).

**Conclusions:**

The perception that pediatric transfers do poorly reflects advanced graft dysfunction in some at the time of transfer. The evidence also suggests that it is not the transfer of care that is the critical issue but rather recipients, somewhere between the ages of 11-14 and 25, are a unique and vulnerable cohort. Effective strategies to improve outcomes across this age group need to be identified and applied consistently.

## Background

The transfer from pediatric to adult care is a challenging area of medicine for adolescents with chronic diseases and especially after kidney transplantation [1]. A high rate of graft loss associated with suspected non-adherence after transfer has been described in a small single center report [2]. It has also been our perception that pediatric patients transferring to our adult center have had a greater rate of graft loss. We also had the impression that non-adherence to medication was a key factor for inferior outcomes and wondered whether differences in patient care after transfer might trigger non-adherence. An alternative explanation for this observation might be that some patients have failing grafts at transfer. However there are several more recent papers that do not support an increased risk of rejection or graft loss in pediatric kidney transplant recipients after their care has been transferred to an adult center. Koshy et al showed stable graft loss rates before and after transfer [3]. Van den Heuvel et al examined acute rejection rates over time and found no increase after transfer [4]. Both studies compared outcomes before and after transfer using patients as their own controls. It is also not clear from these reports whether adolescents transplanted in adult centers suffer the same outcomes as pediatric transplant recipients who transfer to adult care and whether these outcomes are inferior to older recipients.

The purpose of this report was to compare graft survival in young adults transplanted in the pediatric hospital to older adults transplanted in the adult hospital. The null hypothesis was there would be no difference in graft survival. However we anticipated that survival in the pediatric cohort would be inferior. If this was true we would be compelled to change our transfer process.

## Methods

This is a single center program retrospective chart review of consecutive solitary kidney recipients transplanted between January 1990 and December 2009. All recipients who were age ≤35 at the time of transplantation and were from Nova Scotia or Prince Edward Island are included in the analysis. Permission for the study was obtained from our institution ethics review board. Data was abstracted from the program's database and individual patient charts and flow sheets.

Pediatric kidney transplants were performed at the IWK Health Center and adults at the QEII Health Sciences Center (geographically separate sites). However the surgery is performed by the same surgeons. Medical care is provided by pediatric and adult nephrologists at the respective sites. Care is transferred from pediatric to adult medicine at approximately age 18 years. A transfer letter is sent by the pediatric nephrologist to the adult transplant nephrologist along with all relevant records. The pediatric clinic nurse contacts the adult nurse to review the important issues and set the date of transfer. A visit is scheduled within 3 months in the adult clinic.

To test the above hypothesis three cohorts were determined based on age at transplantation and place of transplant. All pediatric (PED) were transplanted in the pediatric hospital. This cohort was compared to 2 older cohorts, who were transplanted in the adult hospital, consisting of young adults (age 18 to < 25, YAD) and older adults (age 25 to 35, ADL),

The status of the patients at transfer was defined at first visit to the adult center. The study examined age at transfer, graft function (serum creatinine), blood pressure, phosphate control, and immunosuppressive therapy. The number of laboratory tests performed was also examined in the year pre and post transfer. We examined the causes of graft loss and defined non-adherence as a contributor to graft loss only if the patient admitted discontinuation of an immunosuppressive medication to an attending physician.

Descriptive statistics are presented as mean and standard deviation or percentages. Differences between groups for variables of interest were performed using parametric and non-parametric tests, where appropriate. To exclude potential differences in technical (more challenging vascular anastomosis) and survival bias (patients with early graft loss less likely to transfer with a functioning transplant) death censored graft survival was examined in patients with functioning kidney transplant at 1 year. Pediatric (PED) graft survival was compared to YAD and ADL using Kaplan-Meier survival tested by log-rank starting at year 1 as time zero. Other covariates were examined for associations with graft survival and these included gender, acute rejection within the first year, donor age, organ source, delayed graft function (need for dialysis), diabetes mellitus at time of transplant, HLA mismatch, peak PRA (%), immunosuppression at transplantation (type of calcineurin inhibitor and type of adjunctive therapy), and recipient and donor CMV status. Cold ischemia time could not be examined given the large amount of missing data. Pre transplant PRA was only examined in deceased donation as this test was not performed on live donor recipients. The Kaplan-Meier method was used to test for significant univariate associations with graft survival. Donor age and peak PRA were also tested by examining tertiles for donor age and 0% versus > 0% for PRA (deceased organ source only). Covariates with a level of significance of < 0.10 were included into the final multivariate Cox model. Significance was tested at the 5% level. Statistical analyses were performed using SPSS 15.0 software (Chicago, IL).

## Results

There were 247 transplants performed in 224 recipients between January 1990 and December 2009. Only 7 (3.1%) were not Caucasian. Table 1 shows the baseline demographics for the three age cohorts. Other than age, diabetes mellitus status and initial immunosuppression, the 3 patient groups were similar at baseline.

**Table 1:** Baseline Characteristics of the Three Age Cohorts at Time of Transplantation

	Pediatric PED N = 56	Young Adult YAD N = 54	Adult ADL N = 137	p-value
Age years	11 ± 5	21 ± 2	30 ± 3	< 0.001
Gender male	29 (52%)	31 (57%)	79 (58%)	0.75
Deceased Donor	31 (55%)	23 (43%)	74 (54%)	0.34
Donor Age years	36 ± 12	37 ± 14	36 ± 15	0.99
Pre-emptive	18 (32%)	15 (28%)	33 (24%)	0.51
1^st ^graft	42 (80%)	45 (83%)	103 (75%)	0.44
CMV D+/R-	12 (21%)	11 (20%)	29 (21%)	0.98
HLA Mismatch	3.5 ± 1.1	3.7 ± 1.4	3.8 ± 1.4	0.35
Delayed Graft Function	8 (14%)	5 (9%)	18 (13%)	0.69
Peak PRA %	4 ± 15	5 ± 15	4 ± 10	0.88
Diabetes Mellitus	0	4 (8%)	20 (18%)	0.007
Rejection within Year 1	22 (39%)	16 (30%)	41 (30%)	0.41
Year of Transplant				
1990-1994	13 (23%)	22 (41%)	45 (33%)	0.055
1995-1999	18 (32%)	9 (17%)	42 (31%)	
2000-2004	14 (25%)	11 (20%)	30 (21%)	
2005-2009	11 (20%)	12 (22%)	20 (14%)	
Immunosuppression				
Individual	25 (45%)	5 (9.3%)	34 (25%)	< 0.001
Depleting Antibody	50 (89%)	38 (70%)	94 (69%)	< 0.001
Cyclosporine (CSA)	4 (7%)	16 (30%)	43 (31%)	< 0.001
Tacrolimus (TAC)	40 (71%)	22 (41%)	50 (36%)	< 0.001
Azathioprine (AZA)	13 (23%)	29 (54%)	75 (55%)	< 0.001
Mycophenolate (MPA)				
Immunosuppression				
Combinations	45 (80%)	21 (39%)	49 (36%)	< 0.001
CSA/AZA	10 (18%)	17 (31%)	35 (26%)	
CSA/MPA	0	1 (1.8%)	1 (0.7%)	
TAC/AZA	4 (7%)	12 (22%)	41 (30%)	
TAC/MMF	1 (1.8%)	3 (5.5%)	11 (8%)	
Other				

**Figure 1:**
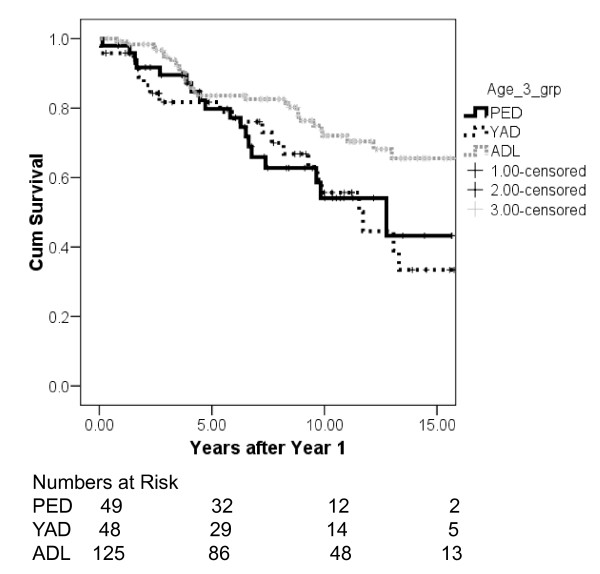
**Death Censored Graft Survival (Kaplan-Meier) after year 1 and stratified by Age Group (PED, Pediatric; YAD, Young Adult age < 25; ADL, Adult age 25-35)**.

Twenty six transplants were lost within 1 year (PED- 7 (12.5%); YAD-6 (11.1%); ADL-13 (9.5%)) and these graft outcomes were excluded. All 7 of the pediatric graft losses occurred at the pediatric center. The remaining were distributed to the PED (n = 49), YAD (n = 48) and ADL (n = 124) cohorts. Figure 1 shows the Kaplan-Meier death censored graft survival for the 3 age cohorts. The survival for the first 2 cohorts (PED and YAD) are almost superimposed where as the graft survival is statistically better (p = 0.04) in the oldest age cohort (ADL; age 25-35). There were 17 patients that died with a functioning graft (PED-2; YAD-2; ADL-13)

In a multivariate Cox model for death censored graft survival, pre-emptive transplantation (HR 0.18, 95% CI 0.07, 0.45, p < 0.001), CMV D+R- status (HR 0.30, 95% CI 0.13, 0.72, p = 0.006) and male gender (HR 0.55, 95% CI 0.36, 0.90, p = 0.017) were associated with much better outcomes whereas acute rejection was associated with an inferior outcome (HR 2.3, 95% CI 1.4, 3.8, p = 0.001). In comparison to the PED group the YAD was statistically similar (HR 0.86, 95% CI 0.44, 1.7, p = 0.66). However the older age category demonstrated better survival (HR 0.45, 95% CI 0.25, 0.82, p = 0.009). HLA mismatch, diabetes mellitus, PRA, immunosuppressive therapy, organ source, and donor age were not significant.

Of the 49 PED cohort 24 transferred to the adult program with a functioning transplant. Of these 24, 18 were followed at this center and the remaining 6 were followed elsewhere. Table 2 shows the characteristics of the patients on transfer to this institution. Of these, 6 had a serum creatinine > 180 μmol/L at transfer and all of these eventually failed. Three of these had advanced dysfunction (serum creatinine > 300 μmol/L) and all failed within 2 years.

**Table 2:** Patient Characteristics at Transfer to Adult Center

	Transfer (n = 18)
Age at transfer	18.6 ± 1.0 (range 16.6-20.9 years)
Gender (male)	9
ESRD	
Congenital	6
Glomerulonephritis	7
Interstitial	3
Other	2
Duration of Function	5.1 ± 3.5 (range 0.9-11 years)
Deceased Donor	11
1^st ^Graft	13
HLA MM	3.5 ± 1.2
Number of patients with rejection pre transfer	7
Donor Age (years)	39 ± 12
Systolic BP	127 ± 11 mm Hg
Albumin	40 ± 3 g/L
Hemoglobin	112 ± 19 g/L
Phosphorous	1.27 ± 0.47 mmol/L
Serum Creatinine	
Year 1 Post Transplant	208 ± 132 μmol/L
At Transfer	182 ± 81 μmol/L
12 months Post Transfer	226 ± 170 μmol/L
Mean Laboratory tests	
12 months pre	28 ± 27
12 months post	15 ± 9
Number of patients with rejection post transfer	5
Mean Follow up	4.92 years
Immunosuppression	
Tacrolimus	10
Cyclosporine	7
Azathioprine	11
MMF	3
Sirolimus	1
Prednisone	18

In the PED group, 4 (21%) of the 19 graft failures were admitted non-adherers whereas the number in the YAD cohort was 3 (19%) of 18 graft losses and in the ADL group was 6 (20%) of 30 graft losses.

## Discussion

The major finding is that there does not appear to be any difference in graft survival for young recipients transplanted at the pediatric center and those young adults transplanted at the adult center. Both groups appear to do worse than a slightly older cohort. This study does not provide any evidence that the transfer from pediatric to adult care is responsible for any increase in graft failure. The perception of early graft loss after transfer likely reflects advanced graft dysfunction in some patients. The more important implication of this report is our conception of the issue which might identify appropriate areas of study. Conceptually, it is not the transfer of care that is the critical issue but rather recipients, somewhere between the ages of 11-14 and 25, are a unique and vulnerable cohort. Effective strategies across this age group need to be identified and applied consistently.

The findings of this study appear to be at odds with a Government of Accountability Office (GAO-07-1117) report of US kidney transplant recipients. In an analysis of the USRDS database of kidney transplant recipients transplanted between 1997 and 2000, researchers also created 3 cohorts of patients (pediatric-1, 218, transition-2, 148 and adult-49, 940) and examined survival [5]. At 5 years post transplantation there were more graft losses in transitional patients (33%) than pediatric (16%) and adult (28%) patients. However, the Scientific Registry of Transplantation Recipients (SRTR) report showed that pediatric recipients between the age of 11-17 have the lower 1, 3, and 5 year graft survival compared to recipients age < 11 and age 18-34 [6]. The GAO study did not perform appropriate survival analyses, and neither the GAO nor the SRTR report tested for statistical significance in an adjusted model and did not distinguish between young adults (age 18-25) and older adults (age 25-35). We suspect that with further analysis of this registry, subjects transplanted within the 18-25 year age will be seen to have death censored graft survival rates that are similar to 11-17 year olds and both will be inferior to graft survival in 25 to 35 year olds. The Canadian study by Koshy et al found relatively constant graft loss rates of about 5-6 per 100 patient years of exposure in age intervals of 14-17.9, 18-19.9 and 20-23.9 [2]. Although some of the recipients transplanted at age 11-17 would have lost their grafts after transfer, the results would also be consistent with the concept that young adults somewhere between ages of 11 and 14 to 25 (the lower threshold is unclear) are a vulnerable cohort rather than the transfer of care being the critical issue.

The literature on transfer to adult care in kidney transplantation is limited. There is a concern that adolescents feel disenfranchised and alienated in the process [7]. Some have advocated a planned transfer that might include the adult nephrologist and nurse visiting the patient in the pediatric center prior to transfer, a joint transition clinic and programmed visits to the adult center prior to transfer [7]. However others have not been able to demonstrate the benefit of a targeted transition clinic [8]. The studies are small but all highlight the challenges of transfer. These and others are concerned about non-adherence [7-9]. It is possible that adolescents and young adults are at a vulnerable time regardless of the initial site of care and whether there is a transfer between centers. Greenstein and Siegal and others have characterized a subgroup of non-adherers as 'invulnerable'. These tend to be younger, less well educated, hide their non-adherence and do not believe missing medication will hurt them [9,10]. Centers caring for adolescent and young adults may need to confront this specific belief.

The study did not find a greater rate of admitted non-adherence as a cause of graft failure despite our initial perception that this might explain observed differences. It is difficult to know the extent of the non-adherence in the entire group since often the admission is only detected at graft failure. No measures of non-adherence have high sensitivities or specificities [11]. We utilized admitted non-adherence with immunosuppressant medications as a specific variable in this analysis, but did not ask all patients in a standardized fashion [12]. We did not evaluate rates of covert non-adherence in this study (for example low or variable drug levels, unfilled prescriptions, etc). It is interesting to note that females had inferior outcomes in comparison to their counterparts. There is some suggestion that body image may play a role in non-adherence in pediatric transplant recipients however there was no clear indication of greater non-adherence in females in this study and gender has not consistently been associated with non-adherence in pediatric transplant recipients [13,14]. Whether some of the difference in graft survival exists between the ADL group and the younger 2 cohorts from undetected non-adherence remains speculative.

Part of the perception that patients do worse after transfer is that some are transferred with failing allografts leading to dialysis within a short time period. Of the cohort transferred almost half have lost their transplant. Age was the reason for transfer, not graft function. Patients were transferred within a narrow age window but at a wide range of times from the date of transplantation and with a wide range of graft function. Many of the patients transferred have significant 'mileage' on their grafts and this is the more likely explanation for the perception of inferior outcomes. Overall the care of the patients at transfer was excellent with well controlled blood pressure and serum phosphate. It is interesting to note that the number of laboratory tests in the year after transfer was about half of the number in the year before transfer. Although there were significant differences in the types of initial immunosuppression between cohorts this did not explain the differences detected. Whether the differences in outcomes could be related to greater immune activity or greater non-immunologic (growth and hormonal changes) stress in younger recipients remain a distinct possibility and should not be discounted.

The study has limitations. To test the hypothesis adequately that transfer of care is the critical event there would need to be a control trial randomizing pediatric kidney recipients at age 18 to continued follow up at the pediatric center compared to transfer and follow up to an adult center. As analyzed in this study a significant difference between the pediatric and young adults could have been missed. A sample size of nearly 700 patients would be required to detect a hazard ratio of 0.80 with a power of 80%. This is a single center Canadian study in a predominately white population with universal health care. Other centers might experience different outcomes. A prospective comprehensive standardized non-adherence study would better detect this as a cause of differences in survival between cohorts than this retrospective analysis. We suspect that non-adherence remains a major explanation but cannot prove it.

Immunosuppression was significantly different between groups at baseline but was not a significant predictor of outcomes. This may have been the small sample size or the fact that many pediatric patients had medication changes, as demonstrated in the composition of drug therapies at transfer (Table 2). The more likely explanation is that the newer therapies have reduced acute rejection rates and improved 1 year graft survival but have not greatly improved survival after the first year [15]. In a recent study by Opelz, no significant survival differences could be detected between recipients treated initially with cyclosporine versus tacrolimus or mychophenolate versus azathioprine [16]. The finding that CMV D+R- recipients fared better was a surprise finding of the study and is largely unexplained. Most studies show that these recipients are at increased risk of both death and graft loss [17]. All patients would have received prophylaxis at both centers. The protocols have evolved over time with IVIG and acyclovir in the early years to the current strategy of oral valganciclovir.

## Conclusions

What this study identifies is that both pediatric patients prior to transfer and adolescents transplanted in the adult center are vulnerable and likely need similar interventions. These might include relatively more immunosuppression, better non-immunosuppressive renal protection strategies, effective education with regards to adherence, and better coping strategies. A large randomized controlled intervention trial would be needed to establish benefit. At present there are likely multiple strategies that might be beneficial and the preferred strategy would need to be established. The study does not negate the fact that adolescents transferring to adult medicine require special attention and expertise, but rather the date of transfer is an artificial time point and focusing at this point may miss opportunities to improve outcomes. Further research in this vulnerable cohort deserves our greater attention.

## Competing interests

The authors declare that they have no competing interests.

## Authors' contributions

All authors participated in the research design, collected data, and data interpretation. All authors read and approved the final manuscript. BK performed the statistical analysis.

## Pre-publication history

The pre-publication history for this paper can be accessed here:

http://www.biomedcentral.com/1471-2369/12/54/prepub
